# Single-cell and bulk transcriptomics reveal a CD8^+^ T-cell gene signature predicting prognosis in diffuse large B-cell lymphoma

**DOI:** 10.3389/fimmu.2025.1685541

**Published:** 2025-12-12

**Authors:** Hengqi Liu, Yingfang Feng, Zhengzi Qian, Zheng Song, Ning Zhang, Jingwei Yu, Xia Liu, Lihua Qiu, Shiyong Zhou, Wenchen Gong, Bin Meng, Hassan Abolhassani, Muhammad Asghar, Lanfang Li, Jin He, Huilai Zhang, Xianhuo Wang

**Affiliations:** 1 State Key Laboratory of Druggability Evaluation and Systematic Translational Medicine/Department of Lymphoma, Tianjin Medical University Cancer Institute and Hospital, National Clinical Research Center for Cancer, Tianjin’s Clinical Research Center for Cancer, Key Laboratory of Cancer Prevention and Therapy, the Sino-US Center for Lymphoma and Leukemia Research, Tianjin, China; 2 Department of Radiation Oncology, Cancer Center, Affiliated Hospital of Xuzhou Medical University; Jiangsu Center for the Collaboration and Innovation of Cancer Biotherapy, Cancer Institute, Xuzhou Medical University, Xuzhou, China; 3 Department of Pathology, Tianjin Medical University Cancer Institute and Hospital, Tianjin, China; 4 Research Center for Immunodeficiencies, Pediatrics Center of Excellence, Children’s Medical Center, Tehran University of Medical Sciences, Tehran, Iran; 5 Division of Immunology, Department of Medical Biochemistry and Biophysics, Karolinska Institute, Stockholm, Sweden; 6 Department of Biomedical Engineering, School of Mechanical and Manufacturing Engineering (SMME), National University of Sciences & Technology (NUST), Islamabad, Pakistan; 7 Department of Biology, Lund University, Lund, Sweden; 8 Department of Sports Sciences and Clinical Biomechanics, University of Southern Denmark, Odense, Denmark

**Keywords:** CD8+ T cells, single-cell RNA sequencing, prognosis, chimeric antigenreceptor T cell therapy, diffuse large B-cell lymphoma

## Abstract

**Background:**

Diffuse large B-cell lymphoma (DLBCL) exhibits immunological heterogeneity that influences outcomes of immunochemotherapy, with CD8+ T cells playing a critical role in patient prognosis.

**Methods:**

We integrated single-cell and bulk transcriptome data to establish a CD8⁺ T cell–associated prognostic signature. Single-cell RNA sequencing data from 29 samples (28 individuals), including DLBCL and reactive lymph nodes/tonsils, were analyzed to characterize CD8⁺ T cell heterogeneity, identify distinct subsets, and screen differentially expressed genes. Least absolute shrinkage and selection operator (LASSO) regression combined with multivariable Cox analysis was applied to bulk RNA-seq datasets to construct a prognostic model.

**Results:**

Analysis of 19,483 CD8⁺ T cells revealed eight transcriptionally distinct subsets, from which 48 genes were associated with clinical outcomes. Eight prognostic genes were incorporated into a CD8⁺ T cell–related signature, with higher CD69 and CD70 expression correlating with inferior survival. The signature effectively stratified patients into high- and low-risk groups that differed in cell-of-origin subtype, mutational landscape, and immune microenvironment characteristics. Moreover, the model showed potential to predict baseline response to chimeric antigen receptor T-cell (CAR-T) therapy.

**Conclusion:**

This study highlights CD8+ T cell heterogeneity in DLBCL and establishes a prognostic gene signature that informs patient survival prediction and CAR-T therapy efficacy.

## Introduction

Diffuse large B-cell lymphoma (DLBCL) is the most common type of malignant lymphoma, accounting for 30% of all non-Hodgkin lymphoma cases (1). While over 60% of patients can be cured with the R-CHOP (Rituximab, Cyclophosphamide, Hydroxydaunomycin, Oncovin, Prednisone) regimen, around 40% of patients experience disease progression during or after initial treatment or relapse following initial remission. The overall survival for patients with relapsed or refractory DLBCL is about 6 months. This indicates that DLBCL exhibits significant clinical and biological heterogeneity, primarily manifested in first histological variations, which have a modest negative impact on prognosis (2). Second, molecular and genetic variations: the cell of origin (COO) classification (3) divides DLBCL into two major subtypes based on gene expression profiling (GEP): germinal center B-cell-like (GCB) and activated B-cell-like (ABC), with 10%-15% of cases remaining unclassifiable. The ABC subtype is associated with a poorer prognosis compared to the GCB subtype (3, 4), and these subtypes exhibit distinct distributions of genetic mutations (5). COO classification helps explain the varying outcomes of R-CHOP or targeted therapies, and further genetic subtypes within these groups have been identified, each with differing prognoses (5–8). Third, tumor microenvironment heterogeneity: The tumor microenvironment surrounding malignant B cells consists of immune cells, stromal cells, blood vessels, and extracellular matrix components, as well as immune cells such as T lymphocytes, macrophages, natural killer cells, and dendritic cells (9). The interactions between tumor cells and their microenvironment contribute significantly to the biological and clinical heterogeneity of DLBCL (5).

Reliable prognostic stratification is crucial for identifying DLBCL patients who are likely to respond to treatment. The International Prognostic Index (IPI) provides valuable prognostic information for patients receiving the R-CHOP regimen. However, for those with relapsed or refractory DLBCL, the IPI, which relies solely on clinical parameters (10–12), does not adequately address tumor heterogeneity or the emergence of immunotherapies. To improve patient classification, additional prognostic factors such as cytogenetics, genomics, and molecular mechanisms need to be considered. Several studies have developed prognostic models based on the immune microenvironment to predict outcomes in DLBCL, particularly in relation to chimeric antigen receptor T-cell (CAR-T) therapy, immunotherapy, and immunochemotherapy (13–16). Immune cell populations, including CD4^+^ T cells, CD8^+^ T cells, regulatory T cells (Treg), myofibroblasts (MFs), macrophages, and dendritic cells, all play significant roles in DLBCL prognosis (16–21). High-throughput single-cell sequencing has proven to be a valuable tool for assessing tumor heterogeneity and understanding the interactions between tumor cells and infiltrating immune cells in the tumor immune microenvironment (22). However, few studies have focused on the prognostic role of CD8^+^ T cell-related genes in DLBCL.

This study utilized single-cell and bulk RNA sequencing data to develop prognostic features linked to CD8^+^ T cell-related genes in DLBCL patients. It assessed survival outcomes, COO classification, genomic alterations, molecular subtypes, tumor immune microenvironment characteristics, and CAR-T therapy efficacy in both high-risk and low-risk patient subgroups.

## Materials and methods

### Data acquisition and processing

DLBCL datasets (GSE181063 (8) and GSE117556 (23)) were obtained from the Gene Expression Omnibus (GEO) (http://www.ncbi.nlm.nih.gov/geo/). RNA sequence transcriptome data of the National Cancer Institute Center for Cancer Research (NCICCR) cohort were downloaded from The Cancer Genome Atlas (TCGA) database (https://portal.gdc.cancer.gov/). The scRNA-Seq data from the study by Steen et al. (14) obtained from the GEO database under the accession number GSE182436 include data from seven cases of DLBCL and one tonsilitis sample. scRNA-Seq from Roider et al. (24) was downloaded from heiDATA (https://heidata.uni-heidelberg.de/) under accession code VRJUNV, including five DLBCLs and three reactive lymph node tissues. Primary central nervous system diffuse large B-cell lymphoma (PCNS-DLBCL) scRNA-seq data were downloaded from Zenodo, including eight samples of seven patients (22). Single-cell RNA sequencing of peripheral blood mononuclear cells (PBMCs) and infusion products from 32 patients treated with CD19 CAR-T therapy was obtained from GEO under accession of GSE197268 (25). The LymphGen classification for DLBCL in the NCICCR cohort was sourced from the original publication (5). The lymphoma microenvironment (LME) subtype information was downloaded from a GitHub repository (26), and the DLBCL classification for the GSE181063 cohort, as defined by Lacy et al., was also obtained from the original publication (8). The data sources mentioned above are summarized in **Supplementary Table S1**. Additionally, we obtained tumor biopsy samples from 162 DLBCL patients from the Tianjin Medical University Cancer Institute and Hospital (TMUCIH) (27). The baseline characteristics of this cohort are summarized in **Supplementary Table S2**. The study was conducted in accordance with the Declaration of Helsinki and approved by the institutional review boards of the Tianjin Medical University Cancer Institute and Hospital (Approval ID: bc20240009). Written and verbal informed consent was obtained from all patients.

### RNA extraction and data processing

RNA extraction and data processing were performed as described in our previous studies (28). Briefly, total RNA was extracted using the RNeasy Kit (Qiagen). The purity of the RNA was evaluated with the NanoPhotometer^®^ spectrophotometer (Implen GmbH, Munich, Germany), and RNA integrity was assessed using the RNA Nano 6000 Assay Kit on the Bioanalyzer 2100 system (Agilent Technologies, Santa Clara, CA, USA). A total of 3μg RNA per sample was used as the input material for RNA sample preparation. The resulting libraries were sequenced on an Illumina platform using 150 bp paired-end reads. Raw data in fastq format were processed through in-house Perl scripts to obtain high-quality clean reads. The reference genome and gene model annotation files were directly downloaded from the relevant genome database. Paired-end clean reads were aligned with Hisat2 v2.0.5 to the hg19 reference genome.

### Dimensionality reduction and clustering of CD8^+^ T cells

The cell type annotations were obtained from the original publications. CD8^+^ T cells were individually extracted from each dataset for subsequent analysis. The count matrix was normalized with default settings, and 2,000 shared top variable genes identified in each of the datasets were scaled and used for principal component analysis (PCA). *Harmony* was applied to remove the batch effects, and the top 30 components were used for downstream analyses. The *RunHarmony* function was applied to correct batch effects among datasets and among samples. The *FindNeighbors* function of Seurat was employed to construct the Shared Nearest Neighbor Graph, based on which unsupervised clustering was performed using the *FindClusters* function in Seurat, with the parameter “*resolution = 0.4*”. For visualization, the dimensionality was further reduced using Uniform Manifold Approximation and Projection (UMAP) implemented in the Seurat function *RunUMAP* with parameter “*dims = 1:30; reduction = harmony*’’. The *FindAllMarkers* function was used to identify cluster-specifically expressed genes (P-value< 0.01 and logFC > 0.25). For any unspecified parameter, we used the default settings.

### Scoring cells using gene expression signatures

**Figure 1:**
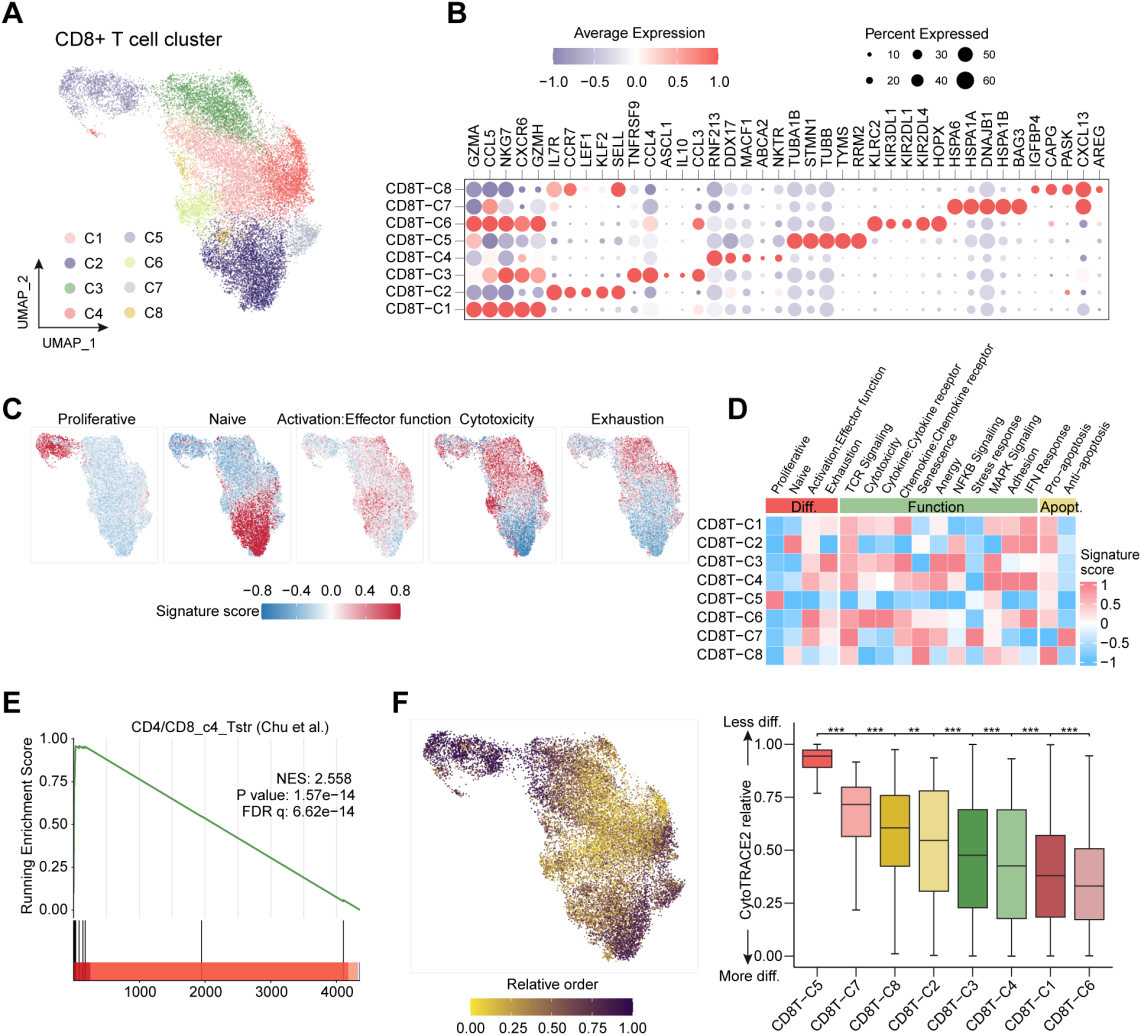
Analysis of CD8^+^ T cell subpopulations in DLBCL based on single-cell gene expression. **(A)** The UMAP projection of CD8^+^ T cells from DLBCL patients, showing the formation of 8 main clusters shown in different colors. The clusters are determined by the gene expression characteristics of each cluster. **(B)** Marker gene expression across defined CD8^+^ T cell clusters. Bubble size is proportional to the percentage of cells expression a gene and color intensity is proportional to average scaled gene expression. **(C)** UMAP plots showing the expression of five signatures of CD8^+^ T cells. **(D)** Heat map illustrating expression of 17 curated gene signatures across CD8^+^ T cell clusters. In **(D)**, ‘Diff.’ and ‘Apopt.’ denote differentiation-related and apoptosis-related signatures, respectively. Heat map was generated based on the scaled gene signature scores. **(E)** Gene Set Enrichment Analysis (GSEA) demonstrated that the CD4/CD8_c4_Tstr gene set (Chu et al.) is enriched in the CD8^+^ T cell subpopulation of our dataset. **(F)** CD8^+^T cell type differentiation map. The left panel shows the relative differentiation trajectory using UMAP, with darker colors indicating lower differentiation levels. The right panel is a boxplot of differentiation potential scores, where higher values indicate greater differentiation potential and lower differentiation levels.

The signature sets of genes for CD8 functional analysis (**Figures 1C, D**) were collected from Chu et al.’s publication (29). The *AddModuleScore* function of *Seurat* was applied with default parameters to score each signature in each cell, and the mean score of all cells in each cluster was calculated. The cluster annotation was determined by combining cluster feature markers and gene expression signature scores.

### Gene set enrichment analysis of cellular stress response state

We applied pre-ranked GSEA using the *clusterProfiler* (30, 31) package (v4.6.2) with 10,000 permutations. The gene signature of “CD4/CD8_c4_Tstr” was obtained from the publication of Chu et al. (29).

### CytoTRACE analysis

To identify the least and most differentiated clusters, we applied CytoTRACE, a computational method that infers the differentiation state of individual cells from scRNA-seq profiles. *CytoTRACE2* (32) R package (v1.0.0) with default parameters was applied to the scRNA-seq datasets without any prior processing.

### Quantification of tissue enrichment of CD8^+^ T cell subsets

The ratio of observed to expected (Ro/e) approach, as previously described (33), was employed to evaluate the enrichment or depletion of individual CD8^+^ T cell subsets across specific tissue types or DLBCL subtypes. In brief, the ratio of the observed cell count to the expected random count was calculated for each cluster using a chi-squared test across various tissue or cancer groups. A Ro/e value greater than 1 indicates enrichment, whereas a value less than 1 signifies depletion of the respective cells in a given tissue or DLBCL subtype.

### Response evaluation

The response of DLBCLs from TMUCIH was assessed visually by the Deauville score (DS), and patients with scores of 1 and 2 were considered to have achieved a complete metabolic response (CMR). A score of 3 was considered as a nearly CMR (nCMR). Scores of 4 and 5 with reduced fluorodeoxyglucose (FDG) uptake compared with baseline indicate a partial response (PR). Scores of 4 and 5 with constant tumor burdens were considered stable disease (SD). Scores of 4 and 5 with increased FDG uptake or the development of new lesions were considered progressive disease (PD).

### Construction of survival-related risk signatures

The genes with logFC > 0.5 and P value< 0.01 in each cluster were used for the construction of survival-related risk signatures. For the NCICCR cohort, we first used univariate Cox regression to identify marker genes associated with survival, and genes with P values < 0.01 were included in subsequent analysis. Subsequently, we performed the Least Absolute Shrinkage and Selection Operator (LASSO) regression to minimize the risk of overfitting and reduce the number of variables. Afterward, multivariate Cox regression was conducted to select the best survival-related variables. In our setting, “covariates” refer to the eight gene-expression inputs used to compute the score. The linear predictor is $Risk\;score=\sum_{i=1}^{n}\beta_{i}x_{i}$ (Cox model without an intercept), where $x_{i}$ is platform-specific log-scale expressions: for RNA-seq cohorts we use $\log_{2}\left(\text{TPM}+1\right)$; for microarray cohorts we use $log_{2}$-transformed normalized intensities. The coefficients $\beta_{i}$ estimated in the discovery cohort are applied unchanged to all validation cohorts. For risk stratification, we dichotomize by the median of Risk score within each cohort rather than carrying over a fixed cut point from the discovery cohort, to account for platform and preprocessing differences.

### Immunofluorescence staining for CD69 and CD70 expression on CD8^+^ T Cells

The paraffin sections were heated at 65 °C for 2 hours, followed by deparaffinization and rehydration, antigen retrieval, and blocking of endogenous peroxidase activity. The sections were then incubated with the primary antibody overnight at 4 °C. The primary antibodies are as follows: CD69 Polyclonal antibody (Proteintech, 10803-1-AP), CD70 Monoclonal antibody (Proteintech, 67749-1-Ig), CD8a Monoclonal antibody (Proteintech, 66868-1-Ig), Anti-CD8 alpha antibody (Abcam, ab93278). The sections were then rewarmed at room temperature, then incubate with the fluorescent secondary antibody. The secondary antibodies are as follows: goat anti-Rabbit IgG (H+L) Cross-Adsorbed Secondary Antibody, Alexa Fluor™ 594 (Invitrogen, A-11012), Goat anti-Mouse IgG (H+L) Cross-Adsorbed Secondary Antibody, Alexa Fluor™ 488 (Invitrogen, A-11001), then washed in PBS for 3 times. Finally, all the sections were stained with Mounting Medium, antifading (with DAPI) (Solarbio,S2110). Formalin-Fixed, Paraffin-Embedded (FFPE) tissue slices from 37 patients diagnosed with DLBCL were analyzed using multicolor immunofluorescence staining to evaluate the expression of CD69 and CD70 on CD8^+^ T cells. CD69 positivity (CD69^+^) was defined as CD69 expression on CD8^+^ T cells>10% of the total CD8^+^ T cell population. CD70 positivity (CD70^+^) was defined as CD70 expression on CD8^+^ T cells>10% of the total CD8^+^ T cell population (34).

### Independent validation of the risk signature

The same formula and coefficients were employed to evaluate the universality of this signature across the independent validation cohorts GSE181063 and GSE117556, as well as an internal DLBCL cohort from our center. Kaplan-Meier curves were similarly generated for these cohorts to assess the predictive capacity of the signature. Furthermore, independent survival analyses were carried out using multivariate Cox regression models.

### Somatic mutation analysis

Mutation information for GSE181063 was retrieved from the original publication, which included targeted deep sequencing of 293 genes. The tumor mutation burden (TMB) was defined as the number of mutations per megabase in the tumor samples. TMB was calculated for each sample, and its association with the risk score was assessed. Group-wise comparisons of mutation frequencies were performed using the *maftools* package (35).

### Tumor microenvironment analysis

The “ESTIMATEScore”, “ImmuneScore” and “StromalScore” were calculated using the *ESTIMATE* package with default parameters (36). Immune-cell fractions were estimated using CIBERSORT (37) with the LM22 signature and 500 permutations; quantile normalization was enabled for microarray cohorts and disabled for RNA-seq cohorts, and analyses were conducted separately within each cohort. Single-sample gene set enrichment analysis (ssGSEA) was performed with the *GSVA* R package *(method = “ssgsea”)* on log2 (TPM + 1) matrices to derive enrichment scores for curated gene sets representing immunosuppressive checkpoints and cytokines. The “IS checkpoints” and “IS cytokines” gene signature were collected from the publication of Kotlov et al. (26).

### Exploring the predictive capability of the risk signature in the CD19 CAR-T therapy cohort

CD19 CAR-T therapy cohort patients were distributed into responder and non-responder subgroups. The differences in the risk scores between different subgroups were determined by the Wilcoxon test.

### Statistical analysis

For prognostic prediction, overall survival (OS) was selected as the study endpoint. OS is defined as the time from the date of diagnosis to the date of last follow-up or death from any cause. Progression-free survival (PFS) was defined as the time from diagnosis to the point of progression or death from any cause. Patients still alive at the end of the study period were censored at the date of last contact. Survival analysis was completed with a Kaplan-Meier (K-M) survival analysis, and differences between groups were analyzed with the log‐rank test. The impact of candidate factors on survival was assessed via univariate and multivariate Cox proportional hazards models. The chi-square method was used to compare categorical variables (Fisher’s test when expected values were< 5). Receiver operating characteristic (ROC) analysis to assess the predictive efficiency of different indices in predicting patients’ outcomes. Unless otherwise specified, P< 0.05 was considered statistically significant. For analyses involving multiple comparisons, adjusted P values were calculated using the Benjamini-Hochberg (BH) method. All the statistical analyses were performed via R software (v4.2.0, http://www.R-project.org).

## Results

### Transcriptional diversity of CD8^+^ T cells in DLBCL

We collected scRNA-seq data from samples of individuals diagnosed with DLBCL and reactive lymph nodes/tonsils, including 29 samples from 28 individuals, totaling 19,483 CD8^+^ T cells. Sample-level clinical information was summarized in **Supplementary Table S3**. Clustering of all CD8^+^ T cells identified eight distinct subsets (**Figure 1A**), each characterized by unique marker gene expression patterns (**Figure 1B**; **Supplementary Table S4**). Using predefined gene sets, we mapped the expression of signatures related to proliferation, naive status, activation/effective function, cytotoxicity, and exhaustion onto UMAP (**Figure 1C**). The proliferative state was primarily associated with cluster C5, while naive cells were mainly found in clusters C2 and C8. Activation/effective function and cytotoxicity were most prominent in cluster C6, and exhaustion-like states were most evident in cluster C3 (**Figure 1C**). Clusters C2 and C8 expressed key naive markers, such as *IL7R*, *CCR7*, *LEF1*, *KLF2*, and *SELL*. Notably, cluster C8 also expressed unique genes, including *IGFBP4*, *CAPG*, *PASK*, *CXCL13*, and *AREG*, along with signals related to cellular senescence and MAPK pathways, in addition to the common markers seen in C2 (**Figures 1B-D**). Cluster C7, a newly identified subset, exhibited stress response characteristics, as indicated by the high expression of the CD4/CD8_c4_Tstr gene set (**Figure 1E**), suggesting that C7 represents a stress-like CD8^+^ T cell population. To elucidate the differentiation trajectory of CD8^+^ T cells, we projected their relative differentiation order onto UMAP (**Figure 1F**). The trajectory began with an initial proliferative state (C5), followed by a stress-like state (C7), naive-like states (C8/C2), exhaustion-like states (C3), and culminated in clusters C4, C1, and C6 (**Figure 1F**).

Based on differentially expressed genes (DEGs) (**Supplementary Table S4**), typical immune markers, and selected gene signatures, we defined seven distinct states: effector memory-like (C1, Tem), naïve-like (C2, Tn-1; C8, Tn-2), exhaustion-like (C3, Tex), NKT-like (C4, NKT-like), proliferative-like (C5, Tprol), NK-like (C6, NK-like), and stress-like (C7, Tstr).

### Relationship between cell subsets and tumor subtypes

**Figure 2:**
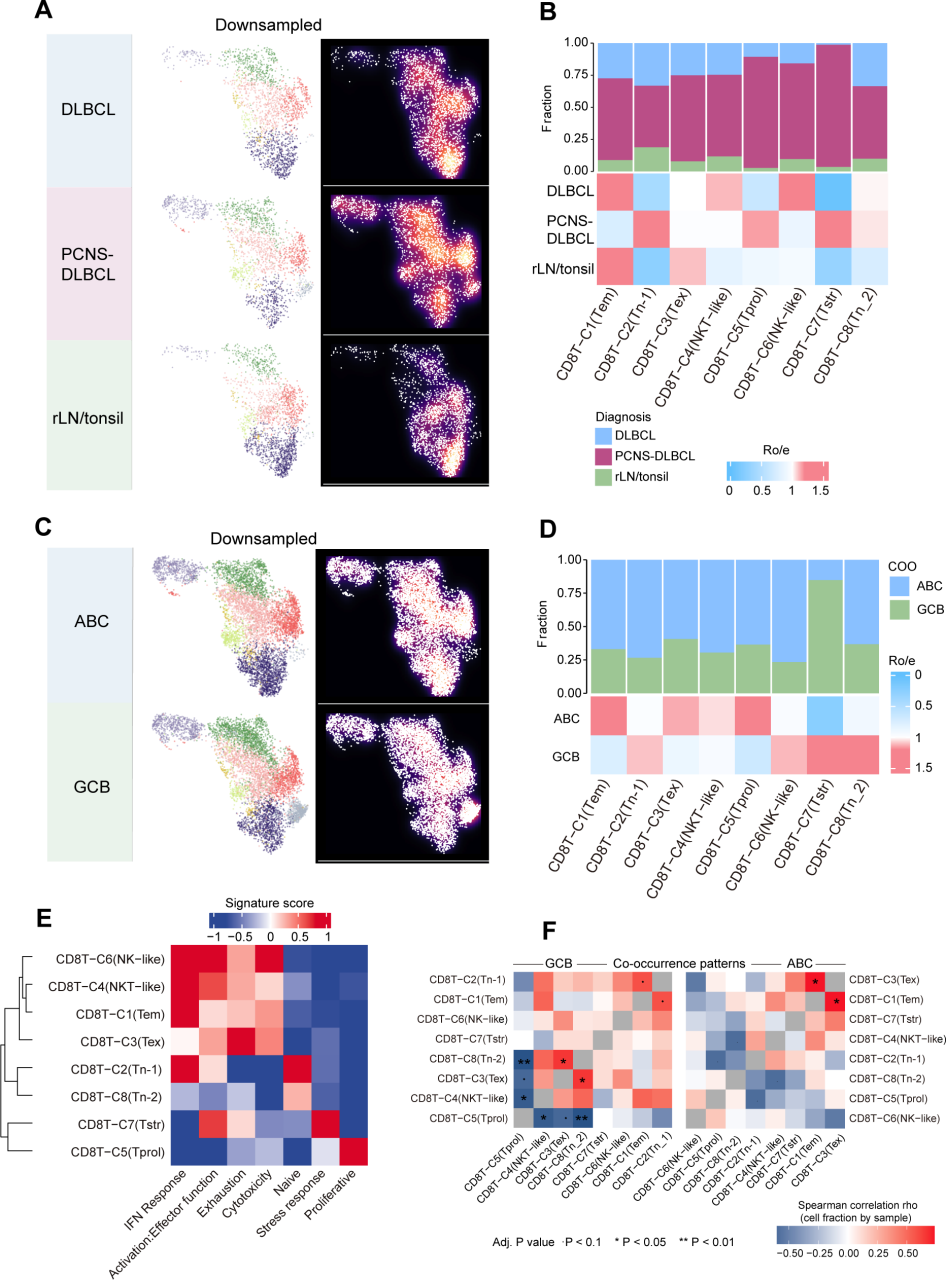
The transcriptional diversity of CD8^+^ T cells. **(A)** UMAP plots of CD8^+^ T cell subpopulations (left) and cell density (right), showing the distribution of CD8^+^ T cells in different tissues. Brighter colors indicate higher density. **(B)** Distribution of CD8^+^ T cell subpopulations in different tissues. The top bar chart shows the relative proportions of each CD8^+^ T cell subpopulation derived from three different tissue types. A heatmap displaying tissue prevalence estimated by Ro/e is presented. **(C)** UMAP plots of CD8^+^ T cell subpopulations (left) and cell density (right), showing the distribution of CD8^+^ T cells across different COO subtypes. Brighter colors indicate higher density. **(D)** Distribution of CD8^+^ T cell subpopulations across different COO subtypes. The top bar chart shows the relative proportions of each CD8^+^ T cell subpopulation derived from two different COO subtypes. A heatmap displaying COO subtype prevalence estimated by Ro/e is presented. **(E)** A clustering heatmap showing the relationship between CD8^+^ T cell subpopulations and seven gene signatures. Red indicates high expression. **(F)** A heatmap showing co-expression among eight CD8^+^ T cell subpopulations derived from GCB and ABC subtype DLBCL. Spearman correlation test,. *P* < 0.1, *P < 0.05, ** *P* < 0.01.

Significant alterations were observed in the CD8^+^ T cell landscape across different tissues. Compared to normal tissues, the proportion of Tex clusters was reduced in DLBCL tissues, whereas the proportions of NKT-like, NK-like, and Tn-2 clusters were increased. In PCNS-DLBCL tissues, the proportions of Tn-1, Tprol, Tstr, and Tn-2 clusters were elevated relative to both normal and DLBCL tissues (**Figures 2A, B**).

DLBCL is classified into molecular subtypes, each characterized by distinct pathogenic mechanisms and prognostic implications. The GCB subtype is generally associated with a more favorable prognosis compared to the ABC subtype. To investigate CD8^+^ T cell changes in DLBCL tissues from different origins, we analyzed the composition of these clusters in the GCB and ABC subtypes. The results revealed that Tem, Tex, NKT-like, and Proliferative clusters were significantly enriched in the ABC subtype, whereas these clusters were either reduced or undetectable in the GCB subtype. Conversely, Tn-1, NK-like, Tstr, and Tn-2 clusters were more abundant in the GCB subtype and were either reduced or undetectable in the ABC subtype (**Figures 2C, D**).

To further characterize relationships among these CD8^+^ T-cell subsets, we first examined how functional programs clustered across the eight clusters. We calculated module scores for gene signatures representing IFN response, activation/effector function, exhaustion, cytotoxicity, naïve state, stress response and proliferation (**Figure 2E**). Hierarchical clustering of these scores showed that IFN response, activation/effector function and cytotoxicity formed a coordinated effector module, whereas exhaustion and stress-response programs clustered together and were often coupled to the proliferative signature. In contrast, the naïve signature segregated from these modules and was largely confined to the Tn clusters. Building on these functional associations, we next asked whether the corresponding subsets tended to co-occur within individual tumors. To this end, we performed Spearman correlation analysis on the eight clusters within the GCB and ABC subtypes (**Figure 2F**). In GCB subtype DLBCL tissues, Tn-2 showed a significant negative correlation with Proliferative (P< 0.01), while NKT-like was negatively correlated with Proliferative (P< 0.05). Additionally, Tn-2 displayed a positive correlation with Tex (P< 0.05). In contrast, in ABC subtype DLBCL tissues, Tex was positively correlated with Tem (P< 0.05).

### Development of the CD8^+^ T cells-correlated risk signature

**Figure 3:**
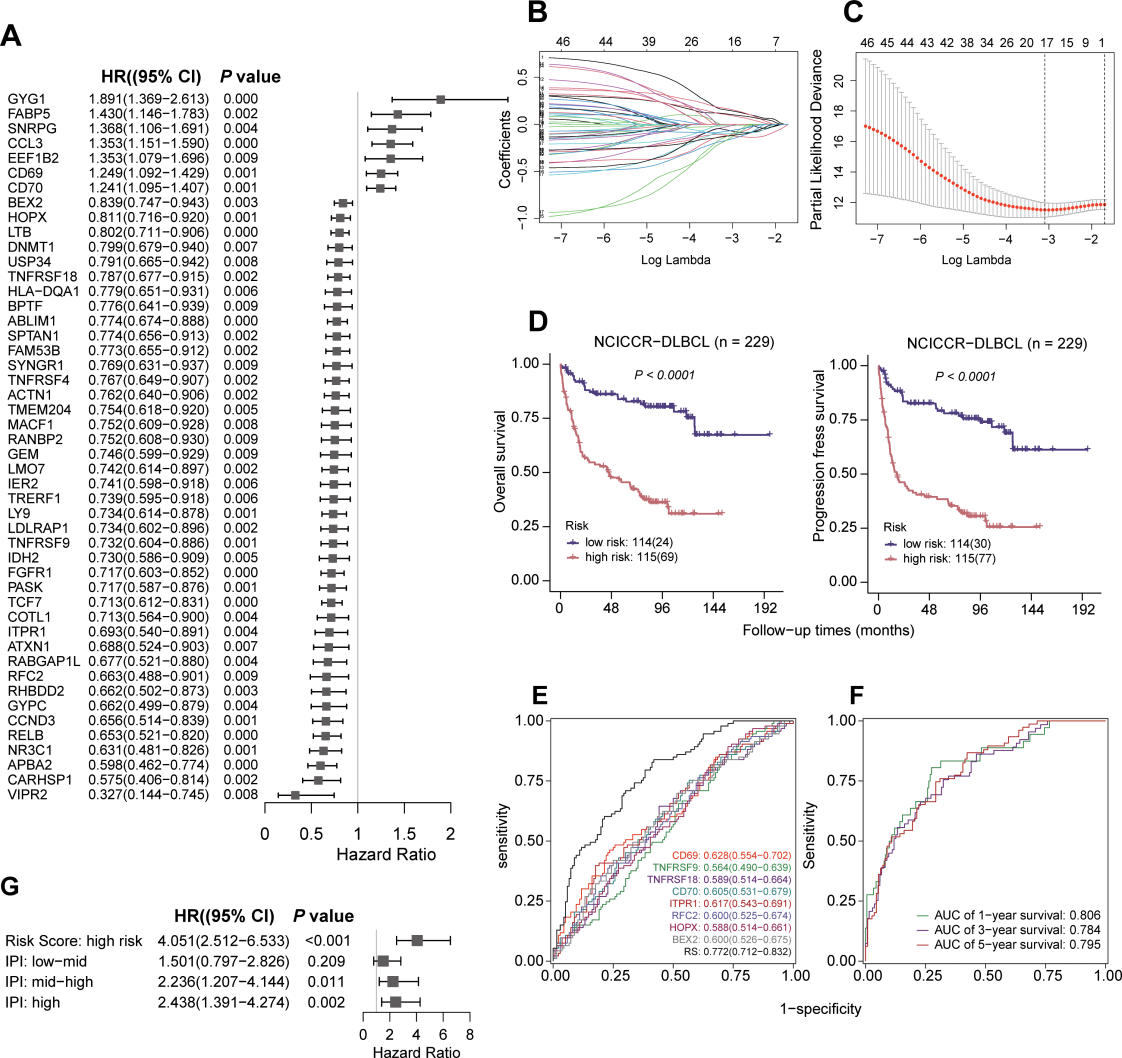
Development of CD8^+^ T cell-related risk signature genes. **(A)** Forest map based on the results of univariate Cox regression analysis. **(B)** The variation characteristics of the coefficient of variables. **(C)** The process of selecting the optimal parameter λ in the Lasso regression model using cross-validation. **(D)** The Kaplan-Meier overall survival (OS) and progression free survival (PFS) curves of the discovery cohort (NCICCR-DLBCL) patients between low risk group (n=114) and high risk group (n=115). NCICCR-DLBCL samples were stratified by risk score. **(E)** ROC curve based on the 8 prognostic feature genes and combined indicators. **(F)** The ROC curve predicts the likelihood of survival for patients at 1, 3, and 5 years in the discovery cohort. **(G)** Univariate Cox regression analysis of Risk Score: high risk, IPI: low-mid, IPI: mid-high and IPI: high in the discovery cohort.

To identify prognostic markers, we first used the CD8^+^ T-cell scRNA-seq dataset to define differentially expressed genes (cluster-specific marker genes) among the identified CD8^+^ T-cell clusters. These CD8^+^ T-cell marker genes were then mapped to the NCICCR DLBCL bulk RNA-seq cohort, in which univariate Cox regression identified 48 genes whose expression levels were significantly associated with clinical outcomes (**Figure 3A**; **Supplementary Table S5**). Based on the bulk NCICCR expression data of these 48 genes, LASSO regression further reduced the set to 40 candidates (**Figure 3B, C**), and multivariate Cox regression finally selected 8 genes (*CD69, CD70, TNFRSF9, TNFRSF18, ITPR1, RFC2, HOPX*, and *BEX2*) to construct a robust prognostic signature (**Table 1**). Based on the median risk score derived from the prognostic signature, DLBCL patients were stratified into high-risk and low-risk subgroups. Kaplan-Meier curves survival analysis demonstrated that the low-risk group had significantly prolonged overall survival (OS) and progression-free survival (PFS) compared to the high-risk group (**Figure 3D**). To validate the predictive performance of the risk score, time-dependent ROC analysis was performed. The risk score exhibited superior predictive accuracy compared to individual genes (**Figure 3E**), with area under the curve (AUC) values of 0.806, 0.784, and 0.795 for 1, 3, and 5-year survival rates, respectively (**Figure 3F**). Furthermore, after adjustment for the IPI score, the risk score remained an independent and effective predictor of poor prognosis in DLBCL patients (**Figure 3G**). These results underscore the critical role of CD8^+^ T cell-related features in predicting clinical outcomes for DLBCL patients, providing a potential tool for improved risk stratification and personalized treatment strategies.

**Table 1:** Multivariate Cox regression analysis confirms 8 genes for constructing a robust prognostic signature.

Gene	coef	HR (95% CI)	P value
CD69	0.278	1.321 (1.108-1.574)	0.002
CD70	0.190	1.210 (1.056-1.385)	0.006
BEX2	-0.150	0.861 (0.759-0.977)	0.021
HOPX	-0.188	0.829 (0.714-0.961)	0.013
TNFRSF18	-0.223	0.800 (0.655-0.977)	0.029
ITPR1	-0.321	0.726 (0.556-0.947)	0.018
TNFRSF9	-0.331	0.718 (0.573-0.901)	0.004
RFC2	-0.432	0.649(0.454-0.927)	0.018

HR, hazard ratio; coef, coefficient; CI, confidence interval.

### Validation of the risk prediction model

**Figure 4:**
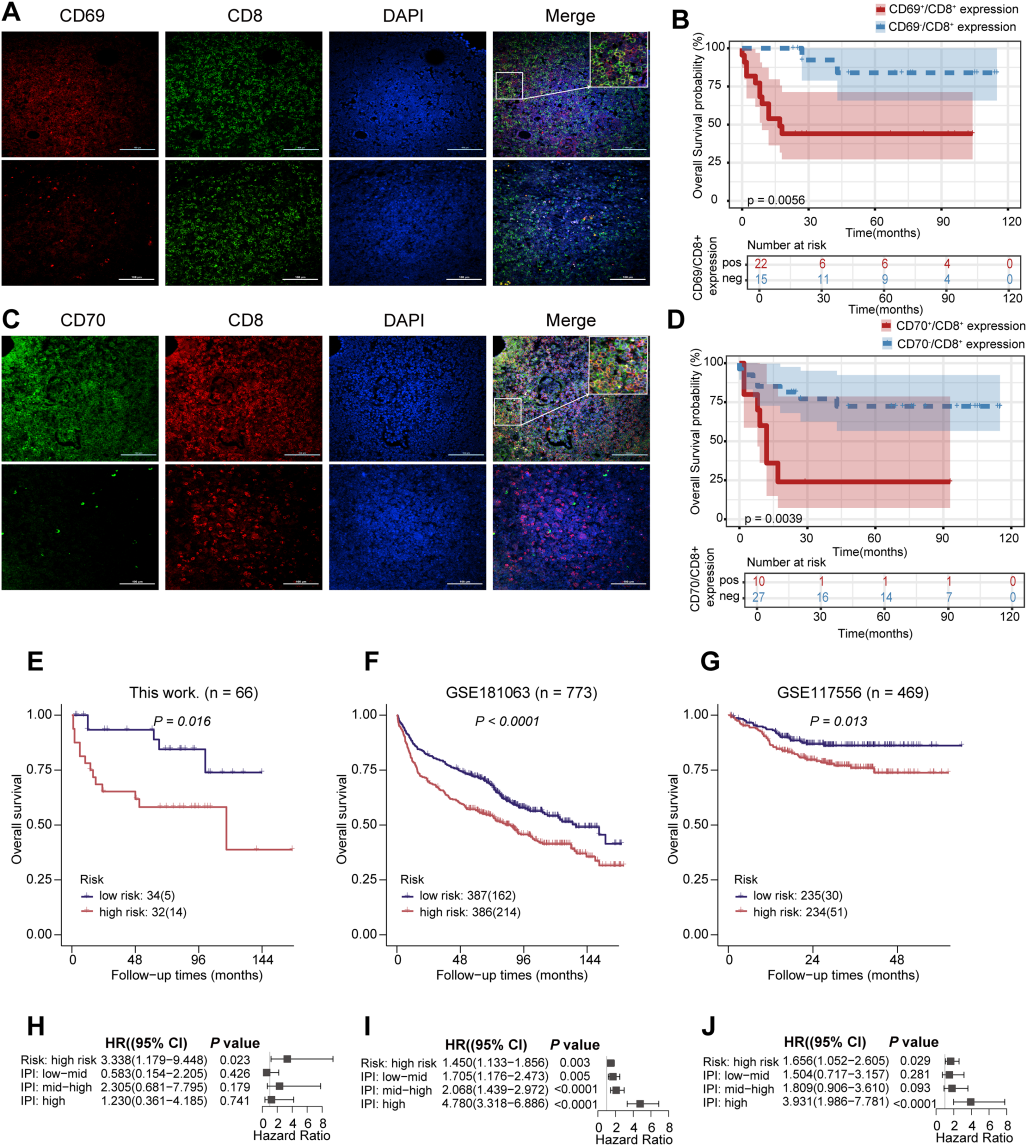
Validation cohort for predicting the risk signature of DLBCL survival based on the discovery cohort. **(A)** Expression of CD69 on infiltrating CD8^+^ T cells in DLBCL (400X). CD69 (red), CD8 (green), DAPI (blue). **(B)** Kaplan–Meier curves of OS in DLBCL patients with CD69^+^/CD8^+^ and CD69^+^/CD8^+^. Cases were classified as CD69^+^/CD8^+^ when ≥10% of infiltrating CD8^+^ T cells expressed CD69. **(C)** Expression of CD70 on infiltrating CD8^+^ T cells in DLBCL (400X). CD70 (green), CD8 (red), DAPI (blue). **(D)** Kaplan–Meier curves of OS in DLBCL patients with CD70^+^/CD8^+^ and CD70^+^/CD8^+^. Cases were classified as CD70^+^/CD8^+^ when ≥10% of infiltrating CD8^+^ T cells expressed CD70. **(E)** The Kaplan-Meier OS curve of the validation cohort (this work. (n=66)) patients between low risk group (n=34) and high risk group (n=32). This work samples were stratified by risk score. **(F)** Univariate Cox regression analysis of Risk Score: high risk, IPI: low-mid, IPI: mid-high and IPI: high in this work. **(G)** The Kaplan-Meier OS curve of the validation cohort (GSE181063 (n=773)) patients between low risk group (n=387) and high risk group (n=386). GSE181063 samples were stratified by risk score. **(H)** Univariate Cox regression analysis of Risk Score: high risk, IPI: low-mid, IPI: mid-high and IPI: high in GSE181063. **(I)** The Kaplan-Meier OS curve of the validation cohort (GSE117556 (n=469)) patients between low risk group (n=235) and high risk group (n=234). GSE117556 samples were stratified by risk score. **(J)** Univariate Cox regression analysis of Risk Score: high risk, IPI: low-mid, IPI: mid-high and IPI: high in GSE117556. Log-rank tests were used to derive p-values for comparisons between two groups.

To evaluate the reproducibility of the constructed risk prediction model in forecasting the prognosis of DLBCL, we performed multiplex immunofluorescence staining on paraffin-embedded tissue sections from DLBCL patients. Our analysis revealed that CD69^+^/CD70^+^ CD8^+^ T cells were significantly associated with poor patient prognosis (**Figures 4A-D**). Next, we validated the model using survival data from three independent cohorts: this work (n=66), GSE181063 (n=773), and GSE117556 (n=469). Clinical subtypes for patients in these cohorts were predicted based on the risk model, and survival differences between the high-risk and low-risk groups were evaluated using Kaplan-Meier survival analysis. The results demonstrated that, across all three validation cohorts, the low-risk group had significantly improved OS and PFS compared to the high-risk group, further confirming the robustness and stability of the model (**Figures 4E-J**).

### Clinical characteristics and genomic alterations across different risk groups

**Figure 5:**
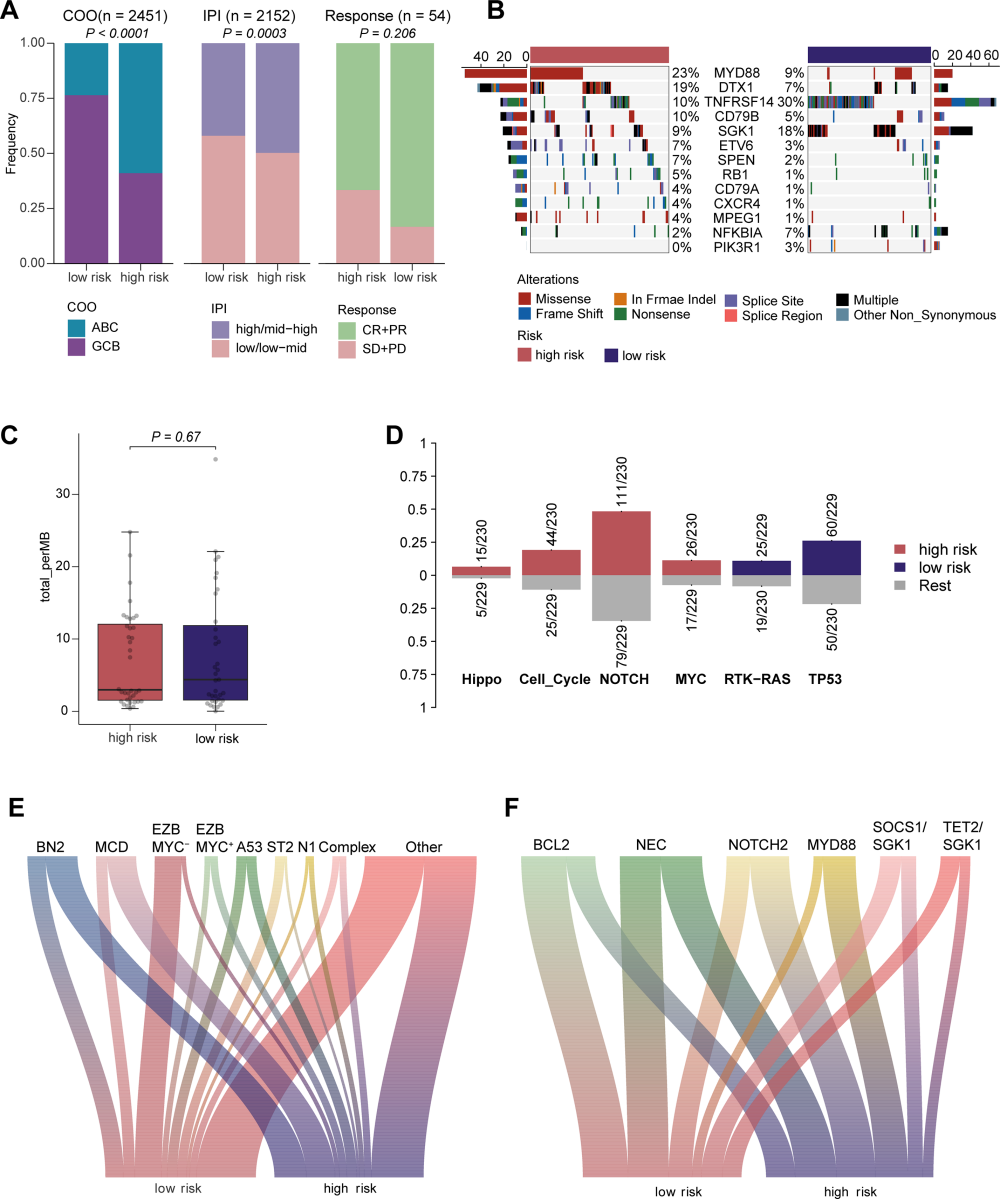
Differences in clinical features and genomic alterations among different risk groups. **(A)** Comparison analysis of differences in COO, IPI, and response between high-risk and low-risk groups. Overall response to first-line R-CHOP/R-CHOP–like therapy in the TMUCIH cohort stratified by risk group. Patients achieving complete or partial response (CR or PR) were grouped as responders (CR+PR), whereas those with stable disease or progressive disease (SD or PD) were grouped as non-responders. **(B)** Mutation status of DLBCL patients based on the risk scores of 8 genes. The sides show the number and frequency of mutations, while the middle displays the gene sample matrix of mutated genes (different colors represent different types of mutations). **(C)** Variations in mutation burden between high-risk and low-risk populations. **(D)** Enrichment of mutant pathways in both groups. **(E)** The Sankey plot illustrates the associations between prognostic risk scores and seven distinct genetic subtypes. **(F)** The Sankey plot illustrates the associations between prognostic risk scores and six distinct genetic subtypes.

Given that COO classification, IPI score, and treatment response are all associated with the prognosis of DLBCL patients, we examined the relationship between risk scores and major clinical features of DLBCL using chi-square tests. The analysis revealed distinct differences in COO distribution between the two risk groups. The high-risk score group was predominantly composed of ABC-DLBCL, while the low-risk score group consisted mainly of GCB-DLBCL. A significant positive correlation was observed between high-risk scores and elevated IPI scores. Furthermore, we calculated the risk scores for the internal cohort (TMU-CIH) and found that the low-risk group had a higher response rate to first-line R-CHOP/R-CHOP-like therapies (**Figure 5A**).

Next, we compared genetic mutation frequencies between the high-risk and low-risk groups. In the high-risk group, the *MYD88* somatic mutation was the most frequent (23%). Thirteen high-frequency differential mutations were observed, from which *MYD88, DTX1, CD79B, ETV6, SPEN, RB1, CD79A, CXCR4*, and *MPEG1*, all of which were enriched in the high-risk group. In contrast, mutations in *TNFRSF14, SGK1, NFKBIA*, and *PI3KR1* were more prevalent in the low-risk group (**Figure 5B**; **Supplementary Table S6**). However, no significant differences in tumor mutation burden (TMB) were detected between the two groups (**Figure 5C**). Additionally, mutations observed in the high-risk group were associated with dysregulation in the Hippo, Cell_Cycle, NOTCH, and MYC pathways, whereas alterations linked to RTK-RAS and TP53 pathway dysregulation were enriched in the low-risk group (**Figure 5D**; **Supplementary Table S7**).

DLBCL constitutes a biologically heterogeneous malignancy, in which molecular subclassification based on genetic hallmarks is indispensable for refined prognostic stratification and the rational design of precision therapeutic interventions. To delineate the relationship between molecular subtypes and prognostic risk scores, we performed a comparative assessment of the LymphGen classification (5) relative to the calculated risk stratifications. According to the original LymphGen framework, MCD and N1 are ABC-type subtypes defined by co-occurring MYD88^L265P^/CD79B mutations or NOTCH1/ID3/BCOR mutations and are associated with poor prognosis; EZB and BN2 are GCB or mixed ABC/GCB subtypes characterized by EZH2 mutations with BCL2 translocations or BCL6 fusions with NOTCH2 mutations and generally show better outcomes; A53 is a mixed subtype with TP53 pathway alterations and adverse prognosis; and ST2 is a predominantly GCB subtype enriched for mutations in epigenetic regulators and PI3K/JAK/STAT signaling and is associated with the most favorable survival. This comparison demonstrated that subtypes linked to an adverse prognosis, including BN2, MCD, N1, and EZB(MYC^+^), were significantly overrepresented within the high-risk group, whereas subtypes associated with a more favorable prognosis, including EZB(MYC^+^) and ST2, were predominantly represented within the low-risk patients. (**Figure 5E**; **Supplementary Table S8**). These results align with those observed in **Figure 5B**, where MYD88 and CD79B, markers of the MCD subtype, were enriched in the high-risk group. Similarly, according to the classification by Stuart et al. (8), the high-risk patients were enriched with the poor-prognosis MYD88 and NOTCH2 subtypes, whereas the low-risk patients were predominantly characterized by the BCL2, SOCS1/SGK1 and TET2/SGK1 subtypes (**Figure 5F**; **Supplementary Table S9**). Consistent with the findings in **Figure 5B**, genetic markers such as *MYD88*, *CD79B*, and *ETV6* (indicative of the MYD88 subtype) and *SPEN* (marker of the NOTCH2 subtype) were enriched in the high-risk group. These results further support the reliability of the predictive model from the perspective of genetic alterations.

### Immune microenvironment differences across risk-stratified populations

**Figure 6:**
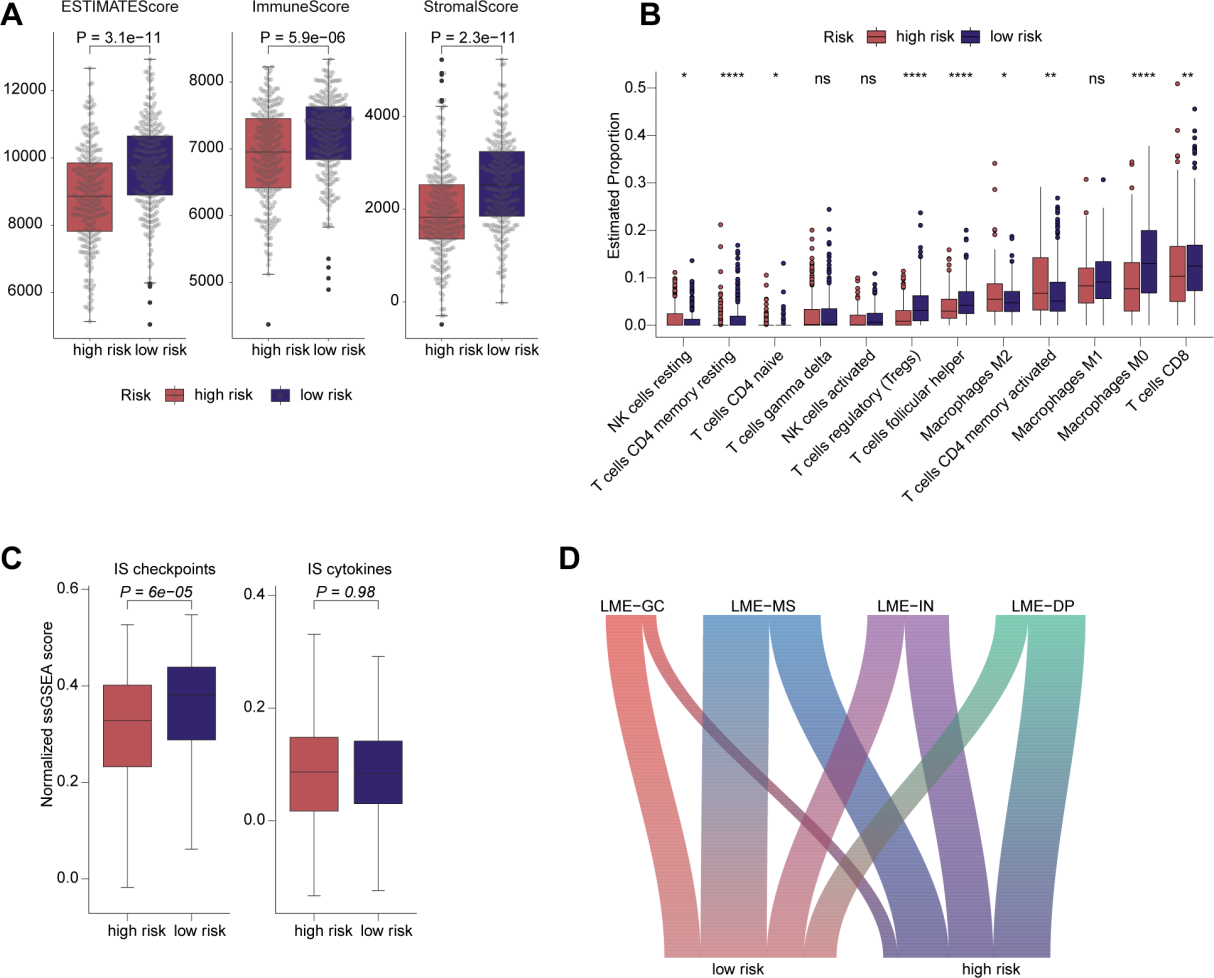
Differences in immune microenvironment among different risk groups. **(A)** The low-risk group exhibited elevated ESTIMATEScore, ImmuneScore, and StromalScore. **(B)** CIBERSORT algorithm was employed to assess the immune infiltration in both groups. **(C)** Comparison of immunosuppression (IS) checkpoints and IS cytokines between the two groups. **(D)** The Sankey chart showing the association of prognostic related risk scores with the four LME categories.

We next assessed the relationship between the risk score from our model and the tumor immune microenvironment in DLBCL. Compared to the low-risk group, the high-risk group exhibited significantly lower ESTIMATE, Immune, and Stromal Scores, indicating higher tumor purity and reduced infiltration of immune and stromal cells in the tumor microenvironment (**Figure 6A**). To further investigate the immune cell composition, we performed immune analysis using the CIBERSORT algorithm. In the low-risk group, the abundance of memory resting CD4^+^ T cells, naive CD4^+^ T cells, Tregs, follicular helper T cells, M0 macrophages, and CD8^+^ T cells was significantly higher, while resting NK cells, M2 macrophages, and memory activated CD4^+^ T cells were significantly reduced (**Figure 6B**). Additionally, we utilized ssGSEA to evaluate the immune status of both groups. This analysis revealed upregulation of immune-suppressive (IS) checkpoints in the low-risk group, though there were no significant differences in the expression of IS cytokines between the two groups (**Figure 6C**). Furthermore, we explored the lymphoma microenvironment (LME) (26), which is closely associated with immune therapy response in DLBCL. Germinal-Center-like LME (LME-GC) and MEsenchymal LME (LME-MS) were notably enriched in the low-risk group, while INflamed LME (LME-IN) and DEpleted LME (LME-DP) were predominantly found in the high-risk group (**Figure 6D**; **Supplementary Table S10**).

### Prediction of CAR-T efficacy based on the model

**Figure 7:**
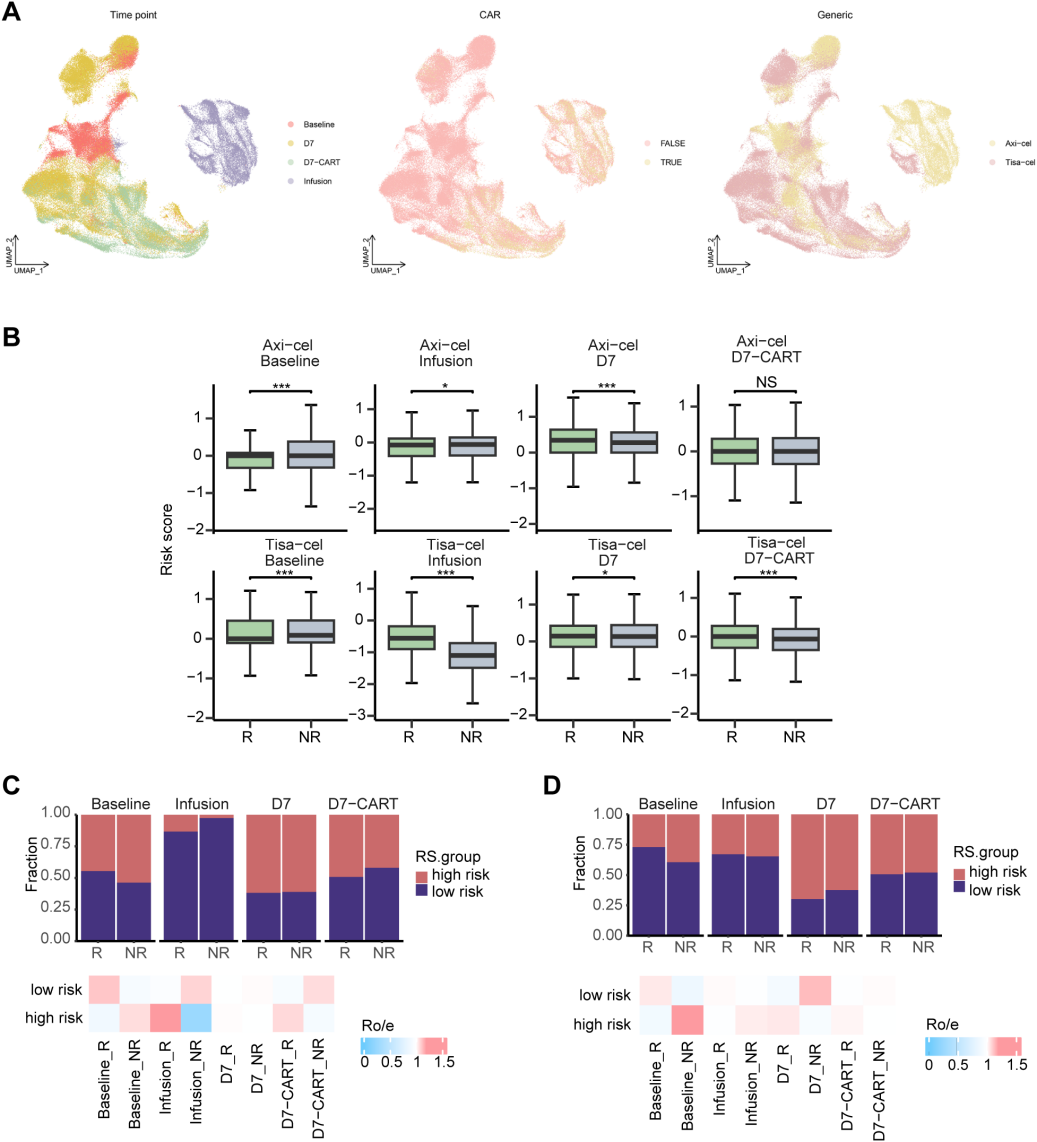
Efficacy of CAR-T therapy across various risk groups. **(A)** UMAP representation of CD8^+^ T cells in the CAR-T dataset. On the left, colors are differentiated by time points. Baseline represents CD8^+^ T cells in baseline PBMC; D7 represents CAR- CD8^+^ T cells in PBMC at 7 days post-CAR-T infusion; D7-CART represents CAR^+^ CD8^+^ T cells in PBMC at 7 days post-CAR-T infusion; infusion represents CD8^+^ T cells in the CAR-T product. In the middle, colors are differentiated based on the presence of CAR structure. FALSE indicates CAR-; TRUE indicates CAR +. On the right, differentiation is based on different CAR-T products. Yellow indicates Axi-cel (n=19); pink indicates Tisa-cel (n=13). **(B)** Risk scores for response to CAR-T (responders, R; nonresponders, NR) at different time points for different products. Student’s t-test, *P<0.05, ** P<0.01, *** P<0.001. **(C)** The distribution of response to CAR-T therapy at different time points in high-risk and low-risk groups of LBCL patients receiving Tisa-cel treatment, based on model risk scores. The top bar chart shows the relative proportions of responses to CAR-T therapy at different time points for different risk groups. **(D)** The distribution of response to CAR-T therapy at different time points in high-risk and low-risk groups of LBCL patients receiving Axi-cel treatment, based on model risk scores. The top bar chart shows the relative proportions of responses to CAR-T therapy at different time points for different risk groups.

CAR-T therapy has revolutionized the treatment of hematologic malignancies; however, only about 50% of patients benefit from this treatment. To evaluate whether our model can predict CAR-T efficacy, we analyzed a scRNA-seq dataset consisting of PBMCs from LBCL patients who received either Axi-cel (n=19) or Tisa-cel (n=13) treatment, along with matched CAR-T products (25). In this dataset, we isolated and clustered all CD8^+^ T cells. The analysis revealed significant differences in the composition of CAR-T products (Infusion) compared to baseline cells (Baseline), D7 CAR-T-negative (D7 CAR-T^−^) cells, and D7 CAR-T-positive (D7 CAR-T^+^) cells. As expected, cells with CAR structures were predominantly found in the Infusion and D7-CAR-T groups, while cells without CAR structures were mainly observed in the Baseline and D7 groups. Additionally, differences in CAR-T product composition were observed between the two products (**Figure 7A**). We then assigned risk scores based on treatment efficacy for samples from various time points across the two CAR-T products. The results showed that, in the Axi-cel cohort, Baseline and Infusion risk scores were higher in the non-responder (NR) group compared to the responder (R) group. In the Tisa-cel cohort, the Baseline risk score was higher in the NR group (**Figure 7B**). Next, we assessed the correlation between the model’s high and low-risk groups and CAR-T efficacy. In the Tisa-cel cohort, the low-risk group at baseline was positively correlated with better treatment efficacy (**Figure 7C**). Similarly, in the Axi-cel cohort, the low-risk group at both Baseline and Infusion showed a positive correlation with improved efficacy (**Figure 7D**). In conclusion, our model’s risk score can predict CAR-T treatment response at baseline, providing valuable insights for the clinical management of DLBCL patients.

## Discussion

Although many DLBCL patients achieve complete remission after first-line R-CHOP treatment, the prognosis for R/R DLBCL is generally poor (1). To improve outcomes for R/R DLBCL patients, treatments like autologous hematopoietic stem cell transplantation, CAR-T therapy, and novel approaches such as targeted apoptosis, B cell receptor pathway inhibitors, and epigenetic regulators have shown promising results. Tumor-infiltrating CD8^+^ T cells, which are associated with better survival, highlight the significant potential of anti-tumor immunity. However, classification studies focusing on CD8^+^ T cell immune function in DLBCL remain limited. scRNA-seq has become an invaluable tool for transcriptional classification of cell types in various cancers (38–40). By analyzing tumor samples, scRNA-seq can identify distinct subsets of T cells and their functional states, revealing mechanisms of immune evasion and pathways related to tumor progression (14). Additionally, scRNA-seq enables the identification of key prognostic genes, providing new biomarkers and potential therapeutic targets (41).

In this study, we analyzed DLBCL scRNA-seq data from the GEO database to characterize CD8^+^ T cell subpopulations. Our analysis identified eight distinct CD8^+^ T cell subgroups, each defined by specific gene markers that could serve as unique identifiers within larger datasets. Using univariate and multivariate Cox regression, along with Lasso regression analysis, we identified eight genes with independent prognostic value and developed a robust prognostic model for DLBCL. Based on this model, NCICCR-DLBCL patients were stratified into low-risk and high-risk groups. Kaplan-Meier survival analysis revealed significantly poorer survival outcomes in the high-risk subgroup. ROC curve analysis demonstrated that the model exhibited moderate sensitivity and specificity in predicting DLBCL outcomes (AUC > 0.7). Furthermore, the model’s accuracy was validated across three external cohorts, yielding consistent results with those observed in the discovery NCICCR-DLBCL cohort.

The prognostic signature developed in this study comprises eight genes: *CD69, CD70, TNFRSF9, TNFRSF18, ITPR1, RFC2, HOPX* and *BEX2*. Among these, *CD69* and *CD70* were identified as adverse prognostic factors and were further analyzed using multicolor immunohistochemistry on DLBCL tissue samples. *CD69* is well-established as an early activation marker for various leukocytes, including T cells, NK cells, and B cells (42–44). *CD70*, a type II transmembrane glycoprotein, is highly expressed in numerous tumors, including hematologic malignancies (45–47). Preclinical and (48–50) clinical studies have demonstrated the efficacy of anti-CD70 monoclonal antibodies and CD70-targeted CAR T cells, with promising outcomes in eradicating CD19^−^ B-cell lymphomas (51) and achieving long-term complete remission in patients with relapsed central nervous system lymphoma through combined CD70- and CD19-CAR T-cell therapy (52). These findings highlight the potential of *CD69* and *CD70* as targets for immunotherapy in DLBCL. The remaining six genes in the prognostic model—*TNFRSF9, TNFRSF18, ITPR1, RFC2, HOPX*, and *BEX2*—serve as positive prognostic indicators. *TNFRSF9* encodes CD137, a co-stimulatory receptor on activated T cells and NK cells, which is critical for immune memory formation and effector function regulation. Agonistic monoclonal antibodies targeting CD137 have shown strong anti-tumor effects in various malignancies, including lymphoma (53). *TNFRSF18* (GITR) enhances anti-tumor immunity by providing co-stimulatory signals to CD4^+^ and CD8^+^ T cells while inhibiting or depleting tumor-infiltrating Tregs (54). *ITPR1*, a member of the IP3 receptor family, facilitates calcium release from intracellular stores, and its targeting has been shown to enhance NK cell-mediated tumor regression *in vivo* (55). *RFC2* plays a critical role in DNA replication and repair and has been associated with TMB, mismatch repair (MMR), and microsatellite instability (MSI) in multiple cancers. In lower-grade glioma (LGG), high *RFC2* expression correlates with increased infiltration of CD8^+^ T cells, resting memory CD4^+^ T cells, and macrophage subsets, suggesting its role in modulating anti-tumor immunity (56). *HOPX*, an epigenetically silenced tumor suppressor, is critical in inhibiting tumorigenesis across various cancer types (57–59). Similarly, *BEX2* acts as a tumor suppressor in malignant gliomas, contributing to its potential role in anti-tumor immunity (60).

Given that COO classification, IPI score, and treatment response are all well-established prognostic factors in DLBCL, we further investigated the relationship between our prognostic model and both COO classification and IPI risk score. The analysis revealed that the high-risk group predominantly consisted of patients with the ABC subtype, consistent with the well-documented poor prognosis associated with this classification. As DLBCL is a biologically heterogeneous malignancy, subclassifying its subtypes based on genetic characteristics is crucial for prognostic stratification and guiding precision therapies. Notably, the MCD subtype, characterized by hallmark genetic alterations such as *MYD88* and *CD79B* mutations, was significantly enriched in the ABC subtype and the high-risk group in our study. This alignment further validates the robustness of our prognostic model through genetic evidence. The tumor immune microenvironment plays a pivotal role in therapeutic efficacy. Our analysis of ESTIMATEScore, ImmuneScore, and StromalScore revealed that the high-risk group exhibited higher tumor purity and lower immune and stromal cell infiltrations within the tumor microenvironment. Furthermore, CIBERSORT analysis demonstrated that CD8^+^ T cells were significantly enriched in the low-risk group. The lymphoma microenvironment (LME) classification, which is closely related to immune therapy in DLBCL, showed significant enrichment of LME-IN and LME-DP in the high-risk group. These findings further validate the prognostic model from the perspective of the immune microenvironment. Lastly, we assessed the model’s ability to predict the efficacy of CAR-T therapy using a single-cell dataset from CAR-T-treated patients. Our results demonstrated that the risk score effectively predicts treatment response to CAR-T therapy at baseline, underscoring its potential clinical utility in guiding therapeutic strategies for DLBCL patients.

In summary, using scRNA-seq data, we successfully developed a prognostic model for DLBCL consisting of eight DEGs. This model demonstrated robust performance in predicting patient prognosis, assessing tumor immune cell infiltration, and forecasting the efficacy of CAR-T therapy. Despite its potential, this study has certain limitations. The model is in its early stages and is based on retrospective data from a relatively small sample size. Consequently, large-scale prospective clinical trials are required to validate its predictive capability and clinical applicability. We hope that this research provides valuable insights into identifying novel immunotherapy targets for DLBCL. While we performed multiplex immunofluorescence on genes A and B from the model, further validation of the remaining genes is needed. Future foundational studies should focus on elucidating the functional roles of these genes in DLBCL prognosis and their potential as therapeutic targets, thereby advancing our understanding of DLBCL biology and immunotherapy strategies.

## Data Availability

The datasets presented in this study can be found in online repositories. The names of the repository/repositories and accession number(s) can be found in the article/**Supplementary Material**.
